# Parents’ Literacy Beliefs, Home Literacy Activities, and Children’s Early Literacy Skills: Stability and Progress Approaching First Grade

**DOI:** 10.3390/bs14111038

**Published:** 2024-11-04

**Authors:** Deborah Bergman Deitcher, Dorit Aram, Dana Abramovich

**Affiliations:** 1 International Liberal Arts Program, Tel Aviv University, Tel Aviv 6997801, Israel; 2 Constantiner School of Education, Tel Aviv University, Tel Aviv 6997801, Israel; dorita@tauex.tau.ac.il (D.A.);

**Keywords:** home literacy environment, letter knowledge, motivation, phonological awareness, early writing

## Abstract

This study explores the stability and progress of parents’ literacy beliefs and home literacy activities and their relationships with their children’s early literacy skills in their last year of preschool. Participants were 50 preschool children (*M* = 61.44 months) and their parents. Data collection sessions occurred in the family home in the fall and spring, with six months between them. At each time point, parents completed questionnaires regarding their beliefs relating to children’s literacy development, parents’ role in supporting literacy development, and the frequency of home literacy activities. We also evaluated the children’s early literacy skills (letter names, letter sounds, word writing, phonological awareness, and motivation for literacy activities). Results revealed overall stability in parents’ beliefs between the two time points, an increase in home literacy activities, progress in children’s early literacy skills, and greater motivation to engage in early literacy activities. We found a positive relationship between parents’ beliefs and home literacy activities in the fall with their children’s early literacy skills in the spring. Further, the progress in parents’ literacy beliefs between the fall and the spring correlated with their children’s progress in early literacy skills, controlling for parents’ education and children’s age. This study highlights the importance of promoting parents’ literacy beliefs and home literacy activities.

## 1. Introduction

Early childhood is a sensitive time period for children’s development (e.g., [1,2,3]). During this time, children’s early language and literacy skills rapidly progress, forming a solid foundation for future reading and academic skills (e.g., [4,5,6]). According to Bronfenbrenner’s [7,8] bioecological systems model, children develop within a series of nested systems with which they interact, and which interact with each other. These systems reflect proximal and distal influences on the child’s development. Children’s language and literacy development can be viewed as taking place within these systems, with the child at the center, nested within microsystems—the contexts closest to the child that exert more proximal influences on the child [9]. These microsystems include both the home and the preschool settings. The interactions between microsystems and potential intersections between them serve as the mesosystem [7,10]. These systems are nested within the exosystem (such as the parents’ workplace), which has more indirect influences on the child. Finally, these are surrounded by the broader macrosystem, where social and cultural values and customs exert a more distal influence on the child. The current study examined children’s early literacy development over the course of a preschool year, focusing particularly on aspects of the home microsystem, looking at parents’ literacy beliefs and home literacy activities and how they relate to children’s early literacy skills. Usually, studies explore these issues at a single time point or longitudinally from preschool to school (e.g., [11]), but not with children in preschool before starting school. As such, in this study, we aimed to explore the stability and progress of parents’ beliefs and home literacy activities and their relationships with children’s literacy skills in their last year of preschool, prior to starting formal schooling.

### 1.1. Parents’ Literacy Beliefs

The home microsystem serves as an important proximal influence on young children’s literacy development [7,8,9,10]. Parents’ literacy beliefs relate both to their expectations of their children’s literacy abilities and their thoughts regarding their role as supporters of their children’s literacy development. These beliefs are important for their children’s lives, as they can be reflected in their behaviors, the environment that they create for their children, and the nature of their interactions with their children [12,13]. More specifically, the beliefs that parents formulate regarding literacy can help shape the richness of the home literacy environment, the frequency and quality of their literacy interactions and children’s independent literacy activities [14,15,16]. For example, parents who believe that their role is to promote their children’s literacy development expose their children to a richer literacy environment [17] and initiate more literacy practices [18]. Similarly, there is evidence that parents’ beliefs regarding the importance of early literacy skills predict their children’s phonological awareness, orthographic knowledge, and enjoyment of shared book reading [19,20]. Additionally, an intervention that promoted parents’ recognition of the importance of shared book reading with their children improved children’s language skills in terms of their vocabulary, morphological awareness, and concepts of print knowledge, and increased their motivation to continue to read books [21]. It seems apparent that parents’ literacy beliefs relate to the kinds of literacy activities within the home setting. As their children approach school, parents may become more focused on impending academic changes, and their beliefs may also go through changes. While some research has examined parents’ beliefs regarding their children’s transition to kindergarten and related this to their children’s academic outcomes (e.g., [22]), we were unable to find any research that longitudinally examined parents’ literacy beliefs during their children’s preschool years, as the children approach more formal schooling.

### 1.2. Home Literacy Activities

Parents who believe more strongly in the importance of early literacy foster a higher quality home literacy environment (e.g., [23]). The home literacy environment is a multifaceted construct that includes the presence of literacy-promoting materials at home (e.g., books, flashcards, letter magnets, writing materials) and children’s individual and joint literacy activities (shared book reading, playing with a digital application on a smartphone, talking about and playing with letters, and trying to read or write words) [22,24,25]. These activities promote children’s understanding, use, and meaning of the written language [26]. A richer home literacy environment was found to relate to children’s language expression and their reading comprehension [27]. Further, the nature of home literacy activities can contribute meaningfully to children’s early literacy skills development [28,29]. In the current study, we explored how home literacy activities may change over the course of a preschool year and how this relates to children’s early literacy skills.

### 1.3. Children’s Early Literacy Skills

Early literacy relates to the foundational knowledge regarding the language system upon which formal reading and writing develop [30]. In alphabetic languages, these early skills include letter knowledge, phonological awareness, and early writing [31]. These skills are included in most prominent models of literacy development (e.g., [32,33,34]). In this study, we focused on early literacy skills that research has shown to be important precursors to, and predictors of, children’s reading and writing (e.g., [35,36]).

#### 1.3.1. Letter Knowledge

Letter knowledge includes recognizing letter names, their sounds, their graphic form, and the connections between these aspects [37]. Having letter knowledge helps children make a connection between spoken and written language [38]. Studies show that letter knowledge is a good predictor of children’s later reading and writing abilities (e.g., [39,40]), and their reading and writing acquisition in school [41,42]. Hebrew utilizes the acrophonic principle, wherein the letter name starts with the sound that it represents, which helps promote reading and writing [43]. Family activities with letters were found to help promote children’s early literacy skills [37,44]. For example, an intervention program with preschoolers promoting letter knowledge and phonological awareness was more effective at advancing children’s reading acquisition than a program with only phonological awareness [45].

#### 1.3.2. Phonological Awareness

Phonological awareness relates to children’s ability to identify and manipulate the sounds of spoken words [46]. Generally, children are first able to divide words into syllables and then into smaller parts of words, such as sub-syllables, and then to phonemes [47]. Learning to read and write requires being able to both take the words apart in their respective sounds and then integrate the phonemes and letters to write the words [48]. Phonological awareness predicts children’s later literacy skills and reading achievement [49,50]. Additionally, parents were found to be able to promote their children’s phonological awareness, which was associated with an increase in children’s motivation to engage in literacy activities [51].

#### 1.3.3. Early Writing

In literate societies, children are interested in writing before formal exposure to writing or reading (e.g., [52,53]). The information that children have about the writing system changes with age. First, children produce signs that capture the general characteristics of writing, such as the unitization of symbols and linearity. Children then write letters, but they are not associated with the sounds of the target words. Children gradually understand the idea of the script when they begin to represent the sounds in words using phonetically-relevant letters (e.g., [54]). Writing encourages integrating phonological awareness and letter knowledge (e.g., [55]), and adults can help support and scaffold this development [35,39]. The level of children’s writing in the preschool years is a good predictor of early literacy skills (e.g., [56]) and later literacy achievements in school [56,57,58].

### 1.4. Children’s Motivation for Early Literacy Activities

Young children are interested in and motivated to engage in literacy activities [59]. For instance, one study [60] reported that young Turkish children generally had high motivation to learn to read. Similarly, kindergarten children enjoyed reading and writing and perceived the value of these skills [61]. Preschoolers’ interest in literacy was related to their phonological awareness, print knowledge, and vocabulary skills [62]. Parents play an important role in fostering their children’s motivation, serving as models for literacy engagement [63]. It was found that both the home literacy environment and parents’ literacy beliefs related to children’s reading interest [64]. Further, parents’ reading beliefs and the home literacy environment predicted children’s reading motivation [65]. Research indicates that children’s literacy motivation is positively associated with their reading achievement, although a bidirectional relationship between these variables is also indicated [66].

### 1.5. Early Literacy in Israeli Preschools

Aside from the home microsystem, much of children’s literacy development takes place within the preschool context, which is impacted by the broader cultural macrosystem. In Israel, where the study took place, the early education system is primarily free- play oriented [67] and preschool teachers’ general approach is that early literacy has to be acquired mainly through everyday activities. The nurturing of early literacy is reflected in the early education curriculum, which includes five major early literacy domains: alphabetic skills (letter knowledge and phonological awareness), emergent writing and reading, oral language, communication skills, and book immersion [68]. The curriculum emphasizes teachers’ autonomy in selecting the instruction methods and the literacy activities based on their assessment of children’s needs and the preschool’s atmosphere.

Studying the implementation of the early literacy curriculum, Sverdlov et al. [69] found that teachers reported actively promoting all literacy areas. The most frequently reported daily activity in preschools was reading books to children. Interestingly, when asked about their preferences, teachers regarded the advancement of oral language, communication skills, and book immersion as more important than the advancement of alphabet skills and emergent writing and reading. We were interested in learning about the less practiced skills of alphabetic knowledge and writing, and how the home context relates to children’s literacy development over the course of the year as they approach formal schooling.

### 1.6. Current Study

In light of the importance of parents’ beliefs and home literacy activities in shaping young children’s early literacy skills and motivation development, the current study set out to examine preschoolers’ literacy development over the course of a preschool year. This can highlight the stability and progress in these areas and provide greater insight into the potential impact of the home context on preschoolers’ literacy development, as children move towards first grade.

The following research questions and hypotheses were examined in the study:

- In what ways do parents’ literacy beliefs and home literacy activities change from the fall to the spring of their children’s preschool year? As there is little longitudinal research exploring parents’ literacy beliefs and home literacy activities over time, this question remained exploratory.
- How do children’s early literacy skills (letter knowledge, phonological awareness, early writing) and their motivation to engage in literacy activities develop over the course of a preschool year? We expected that children’s early literacy skills would improve over the course of the preschool year. Because of a lack of previous research, we did not have a hypothesis regarding the stability or progress of children’s motivation.
- What is the relationship between parents’ literacy beliefs and home literacy activities and children’s early literacy skills development during the course of one year in preschool?
  ○ We hypothesized that parents’ literacy beliefs and home literacy activities would positively relate to children’s early literacy skills at both time points.
  ○ We hypothesized that progress in parents’ literacy beliefs and home literacy activities would relate to progress in children’s early literacy skills. As parents’ beliefs, home literacy activities, and children’s early literacy skills have been associated with contextual variables such as parents’ education levels and children’s age (e.g., [70,71]), we controlled for these variables when examining the changes over the course of the year.

## 2. Materials and Methods

### 2.1. Participants

The participants were 50 Hebrew-speaking children (30 boys and 20 girls) aged 49 to 75 months (*M* = 61.44, *SD* = 7.23) at the first time point. Their parents were mostly married (47 out of 50), and their ages ranged from 26 to 47 years (*M* = 34.78, *SD* = 5.11) for the mothers and 25 to 47 years (*M* = 37.11, *SD* = 4.61) for the fathers. Regarding education, 33 mothers held an academic degree, while 16 mothers had a high school education; 26 fathers had an academic degree, and 20 fathers had a high school education. Of the responding parents, 46 were mothers and 4 were fathers, demonstrating a predominantly maternal sample.

### 2.2. Parents’ Measures

#### 2.2.1. Demographic Questionnaire

The questionnaire solicited demographic information about parents and children. Parents were asked about their own and their spouse’s age and education, and their children’s age and sex.

#### 2.2.2. Parents’ Literacy Beliefs

The questionnaire included 19 statements that referred to parents’ beliefs regarding the level of literacy knowledge expected from young children (e.g., “A preschooler should know the names of the letters of the alphabet”) and parents’ level of involvement in their children’s early literacy development (e.g., “It is recommended that parents will encourage their children to name letters”). About half of the sentences were phrased positively and half negatively to prevent set responses. Parents responded to the items on a 5-point scale ranging from 1 = I do not agree at all to 5 = I totally agree. After reversing relevant items, the mean score across the statements served as the parents’ literacy beliefs. This questionnaire has been validated in numerous other studies in Israeli samples (e.g., [72,73,74]). Reliabilities in the current study were Cronbach’s α = 0.89 and α = 0.87 at the first and second time points, respectively.

#### 2.2.3. Home Literacy Activities

We identified questions related to literacy activities from previous home literacy surveys (e.g., [75,76]). Questions were designed to capture parental teaching practices, where parents reported working jointly with their children on reading or teaching them to write (e.g., “How often do you help your child with learning letters of the alphabet?”), and independent child practices. In the current study, we focused on three items that related specifically to children’s literacy activities in the home: (1) “The child flips through a book or tells themselves a story”; (2) “The child writes their own name”; (3) “The child copies letters or words”. Parents rated each item in terms of the frequency with which the child engages in the activity on a 7-point scale from 0 = almost never to 7 = daily. Reliabilities in the current study were Cronbach’s α = 0.60 and α = 0.64 at the first and second time points, respectively.

#### 2.2.4. Children’s Literacy Motivation

Modeled on measures of children’s literacy interest and motivation [62], this 13-item questionnaire asked parents about their children’s interest, pleasure, and desire to engage in literacy activities [77]. Items related to early literacy activities connected to letters, words, and books (e.g., “My child likes to listen to stories”, “My child shows interest in words he/she knows”). Parents rated their agreement with the items on a scale from 1 = do not agree at all to 5 = agree very much. The average across the items served as the child’s motivation score, with higher scores reflecting greater motivation to engage in early literacy activities. Reliabilities in the current study were Cronbach’s α = 0.85 and α = 0.84 at the first and second time points, respectively.

### 2.3. Children’s Early Literacy Skills Measures

#### 2.3.1. Letter Names and Sounds

This task has been used in previous studies examining early literacy, with good concurrent validity [29,78]. To assess children’s knowledge of letter names and sounds, the child was shown 26 cards on which the 22 letters of the Hebrew alphabet (not including the final letters) were printed, as well as four pictures (butterfly, flower, house, and tree). The pictures were shown so that children less familiar with the letters would feel comfortable. The child was asked: “What is this letter called?” and “What sound does this letter make?” The child’s response to the first three letters received feedback. The sum of the letters the child identified correctly served as the total score. Reliabilities were Cronbach’s α = 0.94 and α = 0.96 for letter names at the first and second time points, respectively, and Cronbach’s α = 0.96 and α = 0.95 for letter sounds at the first and second time points, respectively.

#### 2.3.2. Phonological Awareness

As with measures around the world e.g., [79], this task assessed children’s abilities to identify and produce the opening phoneme in multiple words [80]. The children were presented with 17 monosyllabic words with a consonant–vowel–consonant structure (e.g., *sir*, which means pot in Hebrew). The initial phonemes of the words represent all consonants with an opening sound in Hebrew. For each word, the child was asked, “What is the smallest sound you hear at the beginning of the word?” The child’s response to the first four words received feedback. In line with research into children’s learning of Hebrew, where the consonant–vowel is a natural unit [81,82,83], responses were scored according to a scale used in previous studies: (0) incorrect answer (e.g., for the word ‘*sir*’ the child says ‘lid’ or ‘I don’t know’); (1) subsyllabic (e.g., for the word ‘*sir*’ the child says ‘si’); or (2) phoneme (e.g., for the word ‘*sir*’ the child says ‘s’). The average score across the 17 words served as the total phonological awareness score. Reliabilities were Cronbach’s α = 0.94 and α = 0.96 at the first and second time points, respectively.

#### 2.3.3. Word Writing

This task [84] has been used in previous studies examining writing in preschool children, with good concurrent validity [85,86]. Each child was presented with four drawings of the following objects and asked to write the word for the object: ‘*berez*’ (BRZ, which means faucet in Hebrew), ‘*tzalahat*’ (CLĦT, which means plate), ‘*magash*’ (MGŠ, which means tray) and ‘*afarsek*’ (AFRSK, which means peach). The selected words represent 15 of the 22 letters of the alphabet and do not include the vowel letters. The instruction to the child was, for example, “Here is a drawing of a plate. Under the drawing, please write the word as best as you can”. The written products were scored using the following scale based on Levin and Bus [54]: 1 = signs that are not letters; 2 = random writing—the child wrote letters that are not related to the word; 3 = basic use of consonants—the child used one suitable consonant with the necessary sound not at random; 4 = intermediate consonant writing—the child used more than one of the consonants of the word, but not all of them; 5 = full consonantal writing with additions or interruptions; 6 = correct writing. Inter-rater reliability for scoring was higher than 90%. The average score across the four words served as the word writing score. Internal reliability between the items was Cronbach’s α = 0.86 and α = 0.95 in the first and second time points, respectively.

### 2.4. Procedure

Ethics approval was obtained by Tel Aviv University. Participants were recruited using a snowball method. Parents signed consent forms before the study began. Data were collected by M.A. students in Education, who visited the participants’ homes at two time points, one in the fall and one in the spring, separated by six months. At each time point, they administered all the children’s early literacy skills measures to the children (letter names and sounds, phonological awareness, word writing). Parents were asked to complete the demographic measure at the first time point and the remaining questionnaires (beliefs, home literacy activities, children’s motivation) at both time points.

## 3. Results

We first present descriptive results of the various study variables. This is followed by *t*-tests and correlations examining the differences and similarities in parent and children’s measures at the two time points (fall/spring). Finally, we show how parents’ beliefs and home literacy activities in the fall related to children’s early literacy skills in the spring and how progress in parents’ beliefs’ and home literacy activities related to children’s literacy progress, controlling for parents’ education and children’s age.

### Descriptive Results

Table 1 presents parents’ beliefs, home literacy activities, children’s literacy motivation, and children’s early literacy skills at the fall and spring time points, along with the *t*-test results of the difference in time and the correlations between the two time points.

As can be seen in Table 1, parents’ literacy beliefs were above average at both the fall and spring time points. That is, parents agreed that their children should know about basic literacy skills in their early years and that parents should be involved in promoting this knowledge. There was a significant positive correlation between their beliefs at the two time points, showing overall stability between them. Home literacy activities increased significantly from the fall to the spring. Parents reported that their children’s motivation to engage in literacy activities was medium–high at the two time points. However, there was significantly higher motivation in the spring compared to the fall, and a significant positive correlation between the measures at the two time points.

In terms of the children’s early literacy skills, the results in Table 1 reveal that children significantly progressed from the fall to the spring across the literacy tasks. In the fall, children identified 11 letters by name, with an additional two letters in the spring. They similarly identified four additional sounds in the spring over the 13 they knew in the fall. In the fall, the children tended to provide a sub-syllable whereas, in the spring, more children were able to say the opening phoneme for more of the words, with a significant increase between the two time points. Regarding word writing, in the fall, the children primarily wrote using random letters that were not connected to the words they were asked to write whereas in the spring, they were able to use partial phonological writing, with a significant increase in this variable as well.

We further examined how parents’ beliefs and home literacy activities in the fall related to children’s early literacy skills and literacy motivation in the spring (see Table 2).

The results shown in Table 2 demonstrate that parents’ stronger literacy beliefs were significantly related to children’s higher literacy skills scores and literacy motivation in the spring (except for letter sounds), and more frequent home literacy activities in the fall were significantly related to higher literacy skills (except for phonological awareness) and literacy motivation in the spring.

Finally, we examined how the progress in parents’ beliefs and home literacy activities related to children’s fall-to-spring progress, controlling for the contextual variables of parents’ education and children’s age. Results are presented in Table 3.

The results further showed a significant positive correlation between parents’ progress in their literacy beliefs from the fall to the spring and their children’s progress in early literacy skills (except for word writing) and motivation for literacy activities, controlling for parents’ education and children’s age. Additionally, an increase in home literacy activities from the fall to the spring was significantly related to an increase in children’s letter sound knowledge and motivation to engage in literacy activities.

## 4. Discussion

Using Bronfenbrenner’s bioecological systems model [7,8] as a framework for examining aspects of children’s literacy development, the current study set out to explore parents’ literacy beliefs, home literacy activities, and how they relate to children’s early literacy skills and motivation over the course of a preschool year, measured in the fall and in the spring, as the children approached first grade. Results showed that, overall, parents’ literacy beliefs were stable across the year, while the home literacy activities increased significantly from the fall to the spring. Children improved in their early literacy skills from the fall to the spring, and there was a significant increase in parent-reported motivation of their children to engage in literacy activities as well. Parents’ beliefs in the fall significantly related to children’s early literacy skills (except letter sounds) and motivation in the spring, and home literacy activities in the fall significantly related to children’s early literacy skills (except phonological awareness) and motivation in the spring. Finally, controlling for parents’ education and children’s age—variables known to relate to parents’ beliefs and the home literacy environment (e.g., [70,71])—the change in parents’ beliefs from fall to spring related to the change in children’s literacy skills (except word writing) and motivation from fall to spring, and the progress in home literacy activities from fall to spring related to the change in children’s letter sound knowledge and literacy motivation from fall to spring.

Overall, parents’ beliefs showed stability between the fall and spring time points. Most existing research has explored the relationship between parents’ beliefs and children’s outcomes at a single time point, with stronger beliefs related to higher early literacy skills [19,20,87]. Our findings seems to indicate that parents develop a belief system, relating to children’s early literacy development and parents’ role, which showed some level of stability over time, even as their children came closer to starting formal schooling. There is evidence that parents’ literacy support practices are stable across shared reading and writing tasks [88,89], different children [90], and even across languages [91]. The current findings seem to extend this “style” to parents’ literacy beliefs.

Despite the overall stability, we found that the change in parents’ beliefs from fall to spring significantly related to children’s progress in literacy skills (except word writing), and to their motivation to engage in literacy activities. That is, children whose parents progressed more in their beliefs from fall to spring showed greater improvement in their early literacy skills and motivation from fall to spring, even when controlling for parents’ education and children’s age. Promoting parents’ literacy beliefs thus takes on added importance in fostering their children’s early literacy skills and motivation.

Studies demonstrated that parents’ beliefs relate to how they construct the home literacy environment—the kinds of literacy materials they have in the home, the activities that they encourage their children to engage in, and the home literacy activities [17,92,93,94]. As such, parents who have a stronger belief in the importance of early literacy development and their role in promoting early literacy create a richer home literacy environment with more materials and more frequent and higher-quality literacy activities and interactions. We found a significant increase in home literacy activities between the fall and the spring. Even though parents’ beliefs remained fairly stable between the two time points, they may have increased the frequency or variety of literacy activities, knowing that their children were soon starting formal schooling. Slicker et al. [95] revealed different profiles of U.S. parents regarding their expectations of their children’s transition to kindergarten and their home activities and how these differentially related to children’s outcomes. More research is needed to further explore the stability or change in parental literacy beliefs and how these relate to stability or change in home literacy activities as their children approach the transition to school.

While there was a significant increase in home literacy activities between the fall and the spring, this was only associated significantly with an increase in parent-reported children’s motivation to engage in early literacy activities and in children’s letter–sound knowledge, controlling for parents’ education and children’s age. Previous research has found that preschool children show high levels of motivation to engage in literacy activities [77,96]. Our findings similarly support this, and also indicate that children’s motivation relates to parents’ beliefs which in turn, is associated with how they construct the home literacy environment [17,92,93,94]. This may also be indicative of reciprocal relationships, where parents with more able children might feel motivated by their children’s achievements and more able children might be more motivated by their parents. This is supported by Georgiou et al. [24] who noted that the direction of the relationships between parents’ beliefs and their children’s reading achievement could not be determined and required more research. While motivation is a complex construct, research indicates that preschoolers’ literacy interest directly related to their development of early literacy skills, such as print knowledge and phonological awareness [62]. Cultivating children’s motivation can play an important role in furthering their early literacy development.

There are a number of limitations to the current study’s results. Parents’ literacy beliefs were self-reported, and as such may be subject to social desirability, especially given the importance of literacy and literacy achievement for schooling. Further, the beliefs were fairly high at the fall time point and, consequently, may not have shown much change by the spring. While the study provides a longitudinal look into parents’ beliefs and home literacy activities over the course of their children’s last preschool year, adding alternative methods could enhance the understanding of what transpires in this time. For instance, conducting in-depth interviews or observations of literacy interactions in the home could provide greater insight. Bronfenbrenner emphasized the interactions between various microsystems and the need to explore some of the processes that develop as part of these interactions [10]. As preschool plays an important role in children’s literacy development, examining the nature of parent––preschool interactions and how these relate to parents’ beliefs and home activities could shed greater light on these contextual processes and how children’s literacy develops within them.

## 5. Conclusions

The current study illuminates the home as an important microsystem within which children’s literacy develops over the course of their final preschool year. Parents’ literacy beliefs and home literacy activities both related to children’s early literacy skills and motivation. Parents’ beliefs showed stability over the course of the year, but when they progressed over the course of the year, their children’s early literacy similarly progressed. As such, it is important to try to promote parents’ literacy beliefs early on in their children’s preschool years. This may also help foster richer home literacy activities, which in turn, relates to improved children’s early literacy skills and motivation to engage in literacy activities. Further research on parents’ literacy-related beliefs, how these relate to home literacy activities, and how these may change over time can continue to contribute to understanding and promoting young children’s early literacy development.

## Figures and Tables

**Table 1:** Parents’ beliefs, home literacy activities, and children’s early literacy in the fall and spring.

	Fall		Spring			
	Range	*M* (*SD*)	Range	*M (SD)*	*t*-Test	*r*
Parents’ beliefs	1–5	3.43 (0.70)	1–5	3.50 (0.60)	0.73	0.75 ***
Home literacy activities	1–5	2.69 (0.95)	1–5	2.96 (0.88)	2.46 **	0.63 ***
Literacy motivation	1–5	3.57 (0.70)	1–5	3.73 (0.62)	1.66 *	0.49 ***
Letter names	0–22	11.14 (7.20)	0–22	13.10 (7.83)	3.08 ***	0.83 ***
Letter sounds	0–22	13.04 (8.16)	0–22	17.30 (7.63)	4.90 ***	0.71 ***
Phonological awareness	0–2	0.90 (0.52)	0–2	1.11 (0.60)	3.29 ***	0.60 ***
Word writing	0–6	2.65 (1.24)	0–6	3.15 (1.67)	2.95 ***	0.70 ***

* *p* < 0.05, ** *p* < 0.01, *** *p* < 0.001.

**Table 2:** Relationships between parents’ beliefs and home literacy activities in the fall and children’s literacy skills in the spring.

		Children’s Spring Scores				
		Letter Names	Letter Sounds	Phonological Awareness	Word Writing	Motivation
Children’s fall scores	Parents’ beliefs	0.33 **	0.21	0.24 *	0.41 ***	0.41 **
	Home literacy activities	0.44 ***	0.25 *	0.02	0.37 **	0.53 ***

Motivation = children’s motivation to engage in literacy activities. * *p* < 0.05, ** *p* < 0.01, *** *p* < 0.001.

**Table 3:** Relations between home fall-to-spring progress (parents’ beliefs, home literacy activities) and children’s fall-to-spring progress, controlling for parents’ education and children’s age.

		Children’s Fall-to-Spring Progress				
		Letter Names	Letter Sounds	Phonological Awareness	Word Writing	Motivation
Home fall-to-spring progress (controlling for parental education and children’s age)	Parents’ beliefs	0.28 *	0.35 **	0.29 *	0.10	0.32 *
	Home literacy activities	0.04	0.29 *	0.05	−0.10	0.35 **

* *p* < 0.05, ** *p* < 0.01.

## Data Availability

Data are unavailable due to restrictions of the ethics review board.
